# Prediction of Intraoperative Fluorescence of Brain Gliomas: Correlation between Tumor Blood Flow and the Fluorescence

**DOI:** 10.3390/jcm10112387

**Published:** 2021-05-28

**Authors:** Artem I. Batalov, Sergey A. Goryaynov, Natalya E. Zakharova, Kristina D. Solozhentseva, Alexandra V. Kosyrkova, Alexander A. Potapov, Igor N. Pronin

**Affiliations:** N. N. Burdenko National Medical Research Center of Neurosurgery of the Ministry of Health of the Russian Federation, 125047 Moscow, Russia; abatalov@nsi.ru (A.I.B.); Sgoraynov@nsi.ru (S.A.G.); NZakharova@nsi.ru (N.E.Z.); akosyrkova@nsi.ru (A.V.K.); Pronin@nsi.ru (I.N.P.)

**Keywords:** glioma, fluorescence, tumor blood flow, ASL perfusion

## Abstract

Introduction: The prediction of the fluorescent effect of 5-aminolevulinic acid (5-ALA) in patients with diffuse gliomas can improve the selection of patients. The degree of enhancement of gliomas has been reported to predict 5-ALA fluorescence, while, at the same time, rarer cases of fluorescence have been described in non-enhancing gliomas. Perfusion studies, in particular arterial spin labeling perfusion, have demonstrated high efficiency in determining the degree of malignancy of brain gliomas and may be better for predicting fluorescence than contrast enhancement. The aim of the study was to investigate the relationship between tumor blood flow, measured by ASL, and intraoperative fluorescent glow of gliomas of different grades. Materials and methods: Tumoral blood flow was assessed in 75 patients by pCASL (pseudo-continuous arterial spin labeling) within 1 week prior to surgery. In all cases of tumor removal, 5-ALA had been administered preoperatively. Maximum values of tumoral blood flow (TBF max) were measured, and normalized tumor blood flow (nTBF) was calculated. Results: A total of 76% of patients had significant contrast enhancement, while 24% were non-enhancing. The histopathology revealed 17 WHO grade II gliomas, 12 WHO grade III gliomas and 46 glioblastomas. Overall, there was a relationship between the degree of intraoperative tumor fluorescence and ASL-TBF (R_s_ = 0.28, *p* = 0.02 or the TBF; R_s_ = 0.34, *p* = 0.003 for nTBF). Non-enhancing gliomas were fluorescent in 9/18 patients, with nTBF in fluorescent gliomas being 54.58 ± 32.34 mL/100 mg/s and in non-fluorescent gliomas being 52.99 ± 53.61 mL/100 g/s (*p* > 0.05). Enhancing gliomas were fluorescent in 53/57 patients, with nTBF being 170.17 ± 107.65 mL/100 g/s in fluorescent and 165.52 ± 141.71 in non-fluorescent gliomas (*p* > 0.05). Conclusion: Tumoral blood flow levels measured by non-contrast ASL perfusion method predict the fluorescence by 5-ALA; however, the additional value beyond contrast enhancement is not clear. ASL is, however, useful in cases with contraindication to contrast.

## 1. Introduction

Guideline recommendations for neuro-oncological operations include maximum tumor resection with minimum risk of functional complications where the use of pre-surgical planning (functional magnetic resonance imaging, tractography), intraoperative mapping, microsurgical techniques and intraoperative optics have been found useful [1,2,3,4,5,6,7,8,9]. Intraoperative fluorescent diagnostics, one of the main methods of intraoperative imaging in the surgery of brain tumors of various histologies, facilitates radical tumor removal [10,11,12]. In addition to increasing the radicality of operations, as shown in the first works of Stummer et al. [13,14], the use of fluorescence allows the detection of anaplastic foci in grade II–III gliomas [15,16].

5-ALA (5-aminolevulinic acid) is one of the most widely used agents in fluorescence diagnostics. The mechanism of the fluorescence with 5-ALA is still unknown in detail; however, it was established that it is connected to the metabolism of the 5-ALA into protoporphyrinogen IX and selective accumulation in glioma cells. This agent is used in glioma surgery for making tumor areas that are otherwise indistinguishable fluorescent during the operation and therefore enabling the surgeon to achieve a higher resection rate. With the use of 5-aminolevulinic acid (5-ALA), the fluorescent effect in low- and high-grade gliomas ranges from 40% in LGG (low grade gliomas) to 95% in glioblastomas [17]. 5-ALA is mostly used in adult glioma surgery.

In clinical practice, the degree of contrast enhancement often dictates the use of 5-ALA. However, the degree of contrast enhancement in regular MRI is an imperfect predictor of fluorescence during surgery [15,16,18,19]. Recently, there has been interest in advanced MRI technologies, such as perfusion technologies. Arterial spin labeling is a perfusion technique that is based on marking water protons in blood with the use of special radiofrequency impulses. This method enables the radiologist to acquire the measurements of TBF (tumor blood flow) without the injection of the contrast agent [20]. Tumor blood flow is an index that represents the amount of blood that flows through a tissue in a period of time. ASL perfusion has been shown to reliably predict the degree of malignancy of brain gliomas [21,22,23,24,25]. Since a higher the degree of tumor malignancy is associated with the 5-ALA fluorescent effect, ASL may be a technique to refine current predictions beyond contrast enhancement only or to replace contrast enhancement scans when contraindicated due to allergies or comorbidities. In the existing literature, we did not find studies devoted to the analysis of the correlation between indicators of tumoral blood flow assessed using ASL perfusion and the fluorescent effect in cerebral gliomas of various degrees of malignancy.

The aim of the study was to assess the relationship between tumoral blood flow measured by ASL and intraoperative fluorescent glow of brain gliomas. Specifically, we also evaluated this in contrast-enhancing and non-enhancing diffuse gliomas.

## 2. Materials and Methods

For this study, after the approval of Hospital Local Ethics Committee, patients with newly diagnosed supratentorial glial tumors were recruited. These patients did not receive any chemotherapy or radiotherapy prior to surgical treatment. All patients who received the diagnosis were subsequently surgically treated between the years of 2011 to 2019 at the Neurosurgery Center named after N. N. Burdenko of the Ministry of Health of Russia.

This study was retrospective. Inclusion criteria in this study were: 1. histological diagnosis of the glioma (grades II–IV); 2. surgical removal with the use of 5-ALA; 3. MRI with arterial spin labeling perfusion in the preoperative period; 4. age > 18 years; and 5. no chemo- or radiotherapy before the surgical treatment.

Before surgery, all patients underwent MRI of the brain by a 3.0 T General Electric Signa HD MRI scanner (GE Healthcare) with an 8-channel head coil. The following pulse sequences were used in the study: T1 FSPGR BRAVO with isotropic 1 × 1 × 1 mm voxel and zero gap before and after intravenous administration of contrast agent (or axial T1 with a slice thickness of 5 mm and a gap between slices of 1 mm before contrast and T1 in 3 projections after contrasting); axial T2 with a slice thickness of 5 mm and a slice gap of 1 mm; axial T2-FLAIR with a slice thickness of 5 mm and a slice gap of 1 mm; diffusion weighted imaging (DWI ASSET)with a slice thickness of 5 mm and a slice gap of 1 mm; and pCASL (pseudo-continious arterial spin labeling).

Cerebral blood flow maps were obtained by processing the data from 3D pCASL (pseudo-continuous marking of arterial spins), which was carried out with the following parameters: 3D FSE, 8 lead spiral scanning with the capture of the entire volume of the brain and subsequent reformats with a section thickness of 4 mm; Field of view(FOV = 240 × 240 mm; matrix 128 × 128, zero filling (ZIP) 512; Time to repeat(TR) = 4717 ms; Time to echo(TE = 9.8 ms; numer of excitations(NEX) = 3; post-marking delay (PLD) = 1525 ms; pixel bandwidth = 976.6 Hz/pixel. Duration of scanning = 4 min 30 sec.

The post-processing of the obtained data was performed using the ReadyView software package (GE Healthcare). To measure the intratumoral blood flow, an area of region of interest (ROI) with an area of 20 mm^2^ ± 10 mm^2^ in the zone with the highest TBF (tumor blood flow) was designated (determined from the color flow maps of blood flow) and drawn. To exclude individual differences in blood flow, we performed a normalization of the TBF index (nTBF) to the value of blood flow in the intact white matter of the centrum semiovale of the contralateral hemisphere. For this purpose, a similar-sized ROI (20 mm^2^ ± 10 mm^2^) was placed in the tumor and in the contralateral centrum semiovale white matter.

To obtain a normalized value, the TBF data were divided by blood flow in the centrum semiovale: nTBF = max TBF/TBF of intact white matter in the centrum semiovale of the contralateral hemisphere.

In all cases, blood flow maps were combined with anatomic imaging (T2, T2-FLAIR, post-contrast T1 after intravenous administration of contrast) using the program NeuroRegistration (GE Healthcare).

Statistical processing was carried out in the R Project program (https://www.r-project.org, accessed on 12 December 2019), and the pROC library was used for the ROC (Reciever operator characteristic) analysis. For statistical analysis, nonparametric methods were used in this work. The group comparison was performed using the Mann–Whitney test. The correlation coefficients were calculated using the Spearman method.

Each patient was informed of the benefits and risks connected with the study and signed an informed consent form.

After obtaining consent and the confirmation of the absence of a significant pathology of the liver and kidneys, patients received an oral solution of 5-aminolevulinic acid hydrochloride (Alasens^®^, State Scientific Center “NIOPIK”, Russian Federation) at a dose of 20 mg/kg 2 h before surgery.

All patients underwent tumor removal where the fluorescent effect of 5-ALA was evaluated on a 2-point scale (fluorescent and non-fluorescent tumors).

Neurosurgical intervention was performed using standard microsurgical techniques using a Zeiss Pentero operating microscope with a BL 400 fluorescence module.

After surgical removal of the tumors, the histological diagnosis was established on the basis of the microscopic examination in accordance with the 2007 WHO classification of CNS tumors with the addition of the 2016 WHO classification [26].

## 3. Results

We included 75 patients, 16 of whom were diagnosed with low-grade glioma (LGG) and 59 with high-grade glioma (HGG) (Table 1). There were 38 men and 37 women aged from 20 to 79 years (median: 53, interquartile span: 40.5–59.5 years). Tumor distribution in the study group is presented in Table 1.

We calculated TBF and nTBF in the LGG and HGG groups (Table 2). Tumor blood flow and normalized tumor blood flow were significantly different between low- and high-grade gliomas (*p*-value < 0.001).

In our study, we found a significant correlation between the degree of intraoperative tumor fluorescence and ASL-TBF (R = 0.28, *p* = 0.02 for the TBF; R = 0.34, *p* = 0.003 for nTBF).

Forty-six percent of diffuse astrocytomas and 50% of oligodendrogliomas in our study did not demonstrate intraoperative fluorescence. Among HGG, the percentage of non-fluorescent tumors was 8.5. All glioblastomas were fluoropositive. The percentage of fluoropositive anaplastic astrocytomas and anaplastic oligodendrogliomas was 61.5.

**Figure 1:**
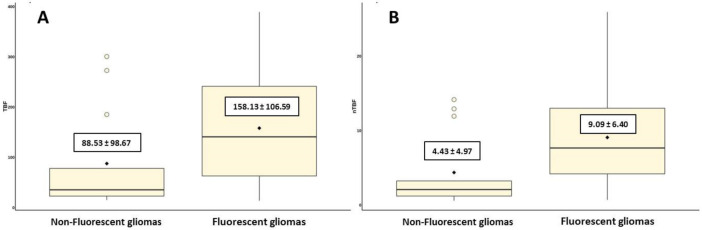
A plot diagram showing blood flow in groups of fluorescent and non-fluorescent gliomas using the values of maximum tumor blood flow (**A**) and maximum normalized tumor blood flow (**B**).

To investigate the possibility of using ASL perfusion for predicting intraoperative tumor fluorescence, we divided all patients into two groups: non-fluorescent tumors and fluorescent tumors. TBF and nTBF values in the group of fluorescent tumors were significantly higher compared to tumors that did not demonstrate intraoperative fluorescence (*p* = 0.016 and *p* = 0.004 for TBF and nTBF, respectively) (Table 3, Figure 1).

**Figure 2:**
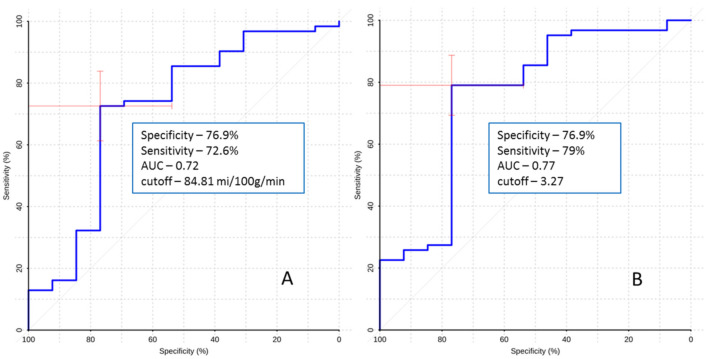
The ROC curve. Comparison of maximum blood flow (**A**) and maximum normalized blood flow (**B**) between fluorescent and non-fluorescent gliomas. AUC—area under curve.

To determine the value of ASL perfusion in predicting intraoperative fluorescence, ROC analysis was performed. The obtained parameters are presented in Figure 2.

The obtained results show that ASL perfusion can be used to predict fluorescence during 5-ALA-guided surgery to some extent. We were able to establish that there is a statistically significant difference in the frequency of occurrence of the fluorescence between tumors with high TBF and tumors with low TBF; moreover, we were able to obtain the cut-off for that distinction. However, the sensitivity and specificity were not high. Therefore, we can conclude that there is a weak correlation between TBF and fluorescence.

Separately, we analyzed the ability of the contrast enhancement to predict fluorescence. We calculated that 71.4% of enhancing tumors showed intraoperative fluorescence, while only 42% of non-enhancing tumors showed fluorescence.

It was discovered that sensitivity for this method was 85.48%, and specificity was 69.23%.

Next, we analyzed the relationship between ASL perfusion, tumor fluorescence and contrast enhancement (Table 4 and Table 5).

As demonstrated above, the optimal cut-off for maximum blood flow using ASL perfusion to predict tumor fluorescence was 84.81 mL/100 mg/min. We observed that nine (14.5%) patients in the group with fluorescent tumors had no contrast enhancement. However, when analyzing the TBF in these patients it was found that only one of these patients had numbers exceeding the cut-off.

In non-fluorescent tumors there were four cases of contrast enhancing tumors. The TBF for these tumors in two cases was higher than cut-off and in two cases lower than cut-off.

**Figure 3:**
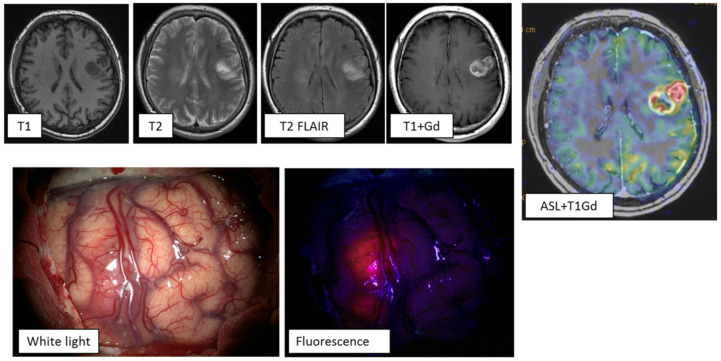
MRI study of a patient with multiple primary glioblastomas. ASL perfusion in the tumor of the left frontal lobe revealed areas of hyperperfusion: TBF = 289.7 mL/100 g/min. Tumor demonstrated vivid contrast enhancement. During surgery using fluorescence diagnostics, this tumor showed an intense glow. Gd—gadolinium.

**Clinical case 1:** A 61-year-old female with symptoms of motor aphasia. MRI study demonstrated a left frontal lobe intracerebral lesion with central necrotic changes and increased pathological contrast enhancement mainly in the peripheral parts of the tumor. ASL perfusion revealed high tumor blood flow (TBF up to 289.7 mL/100 g/min). Another hyperintense lesion on T2 and T2-FLAIR was present in the right basal ganglia without any signs of pathological enhancement. On the basis of the clinical and radiological data, multiple primary glioblastomas were diagnosed (Figure 3). The tumor of the left frontal lobe was removed using intraoperative fluorescence diagnostics. Intraoperatively, the tumor showed intense fluorescence, facilitating radical tumor removal (the entire visible part of the tumor was removed. Histological diagnosis: NOS glioblastoma).

**Figure 4:**
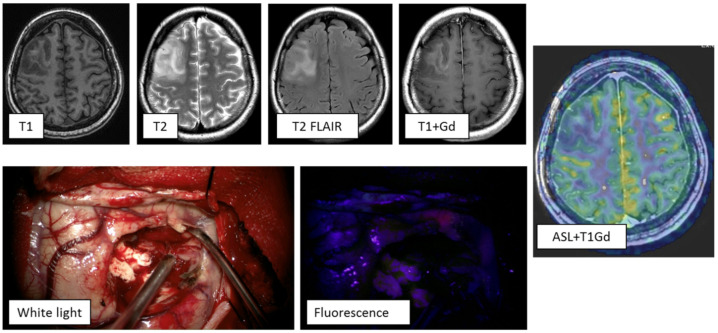
MRI study of a patient with a tumor of the right frontal lobe. ASL perfusion did not reveal areas of increased blood flow in the tumor stroma: TBF = 58.51 mL/100 g/min. During the operation, using fluorescence diagnostics, a dotted area of the fluorescence was visualized in the tumor, which was insufficient to determine its boundaries.

**Clinical case 2:** Patient with a medical history of a single generalized epileptic seizure, a month after which an MRI study was performed. MRI demonstrated a right frontal lobe lesion, hyperintense on T2 and T2-FLAIR and hypointense on T1. Post contrast, enhancement of the stroma of the mass was visualized. ASL perfusion showed low tumor blood flow (TBF up to 58.51 mL/100 g/min). Based on clinical and radiological data, a high-grade glioma was diagnosed (Figure 4). The tumor was removed using intraoperative fluorescence diagnostics. Intraoperatively, the tumor showed weak focal fluorescence, which was insufficient to define the tumor boundaries. Histological diagnosis: Oligodendroglioma with a site of WHO grade III anaplasia and positive expression of IDH 1 (R132H).

## 4. Discussion

In our study we established that ASL perfusion is able to predict the fluorescence to some extent. We obtained the cut-off for it (84.81 mL/100 g/min), specificity (76.9%) and sensitivity (72.6%). In addition to that, we compared the predictive value of ASL perfusion to contrast enhancement and were unable to find that ASL perfusion could add any information. However, this method could be useful in patients in whom contrast is contraindicated.

The influence of various clinical, neuroimaging and pathomorphological parameters on the fluorescent effect was studied [17,18,19,27]. For example, anticonvulsants lower fluorescence, and a higher degree of malignancy leads to a higher fluorescence degree.

The role of various preoperative and neuroimaging factors in predicting the fluorescent effect of 5-ALA in brain gliomas is still unclear.

However, contrast enhancement of the tumor on MRI has been traditionally regarded as a marker of malignant brain gliomas and significantly correlated with the accumulation of 5-ALA in tumor cells. This corresponds to the data that have been previously reported [12,28,29]. Contrast enhancement may be absent in malignant high-grade gliomas, especially in cases of anaplastic astrocytomas [30]. Importantly, there is a possibility of fluorescence in grade II–III tumors that do not demonstrate contrast enhancement on preoperative MRI [13]. Consequently, contrast enhancement of the tumor is a suboptimal predictor of glioma fluorescence during surgery. This has led to a search for other MRI markers that can predict the glow of gliomas during surgery.

One of the most important processes which occurs in glial tumors is neoangiogenesis. High-grade gliomas are characterized by more active neoangiogenesis. We assumed that this fact could be linked to both elevated blood flow measured by ASL perfusion and a fluorescent effect during the operation. We suggested that high vascularity leads to high blood flow and so could help to predict presence of intraoperative fluorescence. However, there is a limitation to this assumption due to the mechanisms of 5-ALA fluorescence. The uptake of 5-ALA is an active process via transmembrane proteins, and, in addition to that, 5-ALA must be metabolized to its active compound protoporphyrin IX. Therefore, the link between high vascularity in glioblastomas and the presence of 5-ALA fluorescence is not direct.

In our work, we compared the predictive ability of contrast enhancement and ASL perfusion. The majority of fluorescent non-enhancing gliomas also had a low TBF, limiting the predictive potential of ASL in predictions beyond the contrast enhancement. However, ASL perfusion is a good approach for predicting 5-ALA’s usefulness in patients with an allergy to gadolinium and severe kidney disease.

Kimberley S. Samkoe et al. [31] showed that although the ALA-5 concentration and gadolinium enhancement are linearly correlated, there appears to be a limit to the ability of MRI to detect diffuse gliomas components, likely due to tumor cell cluster size or amount of blood–brain barrier breakdown. Their study was devoted specifically to the infiltratively growing diffuse glioma components and was conducted on mice. In our work, we were able to discover the same results for glioblastomas: all glioblastomas in our study were fluoropositive, and the majority of them were contrast enhancing, though not all (93.5%).

The results in lower grades of glioma are less conspicuous. In groups II and III combined, 71.4% of enhancing tumors showed intraoperative fluorescence, whereas 42.8% of non-enhancing tumors showed fluorescence.

These results are also consistent with findings in the literature [16,32]. We were not able to find that contrast enhancement or ASL perfusion have a sufficient predictive value in these patients. In the work of Jaber et al. [32], the predictive value of the contrast enhancement was also studied. It was shown that 78% of enhancing tumors showed fluorescence in contrast to 16% of non-enhancing tumors. The results for non-enhancing tumors differ from ours, probably due to the fact that we had a significantly lower number of patients with grade II and grade III tumors.

Threshold values of blood flow were obtained, which allow prediction of the presence of fluorescence of tumors at the preoperative stage with sufficiently high specificity and sensitivity (76.9% and 79%, respectively).

## 5. Limitations of the Study

This is a retrospective study. The limitations of retrospective studies include the heterogenicity of the research groups and the possibility that they have an inferior level of evidence compared to prospective studies. Therefore, it can be concluded that future prospective studies about the correlation between ASL perfusion and fluorescence are required.

The second limitation of this study is the qualitative classification of the fluorescence. This classification could have low interobserver agreement; therefore, future research about the correlation between ASL perfusion and quantitatively measured fluorescence is required.

The third limitation of the study is selection of the patients. In the study group there were no grade I tumors, and there was a low number of grade III tumors. Further studies in these patient groups could be beneficial.

## 6. Conclusions

The use of non-contrast ASL perfusion before surgery makes it possible to predict the absence or presence of a fluorescent effect without the use of contrast. The prediction is in a similar range to contrast enhancement. Contrast enhancement demonstrates higher sensitivity; however, the specificity of the contrast enhancement is lower. However, in our work we were unable to find a higher predictive value of ASL perfusion compared to contrast enhancement. Further studies with a higher number of patients and more advanced and physiological MRI imaging sequences in patients with non-enhancing gliomas are necessary.

## Figures and Tables

**Table 1:** Tumor distribution in the study group.

Histopathological Diagnosis	Grade, WHO	*n*
Gemistocytic astrocytoma	II	2
Diffuse astrocytoma	II	10
Oligodendroglioma	II	4
Anaplastic astrocytoma	III	7
Anaplastic oligodendroglioma	III	6
Glioblastoma	IV	46

**Table 2:** Mean values of tumor blood flow and normalized tumor blood flow in low- and high-grade gliomas.

		Mean	St. Dev.
TBF (mL/100 g/min)	LGG	30.75	13.67
	HGG	177.14	101.05
nTBF	LGG	1.57	0.63
	HGG	10.09	6.05

LGG—Low grade glioma, HGG—high grade glioma, TBF—tumor blood flow.

**Table 3:** Maximum (TBF) and normalized maximum (nTBF) blood flow values in groups of fluorescent and non-fluorescent gliomas.

Presence of Intraoperativefluorescence	Mean Maximum Tumor Blood Flow (TBF) mL/100 g/min	Mean Normalized Maximum Blood Flow (nTBF)
Non-fluorescent gliomas	88.53 ± 98,67	4.43 ± 4.97
Fluorescent gliomas	158.13 ± 106.59	9.09 ± 6.40
Confidence level	*p* = 0.016	*p* = 0.004

**Table 4:** The relationship between contrast enhancement, ASL perfusion and fluorescence.

	Fluorescent Status	*n*	TBF ± St. Dev	*p* Value
Enhancing	Fluorescent	53	170.17 ± 107.65	>0.05
	Non-fluorescent	4	165.52 ± 141.71	
Non-enhancing	Fluorescent	9	54.58 ± 32.34	>0.05
	Non-fluorescent	9	52.99 ± 53.61	

**Table 5:** Contrast enhancement and prediction based upon defined optimal cut-off.

	<84.81 mL/100 mg/min	= or >84.81 mL/100 mg/min
Enhancing	11 (19.3%)	46 (80.7%)
Non-enhancing	15 (83.3%)	3 (16.7%)

## Data Availability

The data presented in this study are available on request from the corresponding author. The data are not publicly available due to the fact that it could contain sensitive information about patients.
